# Characterization of the microRNA408-LACCASE5 module as a regulatory axis for photosynthetic efficiency in *Medicago ruthenica*: implications for forage yield enhancement

**DOI:** 10.3389/fgene.2023.1295222

**Published:** 2023-11-28

**Authors:** Yutong Zhang, Wei Yan, Yu Qiao, Xia Gao, Fang Tang, Cuiping Gao, Fengling Shi

**Affiliations:** ^1^ Key Laboratory of Forage Cultivation, Processing and High Efficient Utilization of the Ministry of Agriculture and Key Laboratory of Grassland Resources of the Ministry of Education, College of Grassland Resources and Environment, Inner Mongolia Agricultural University, Hohhot, China; ^2^ Inner Mongolia Academy of Science and Technology, Hohhot, China; ^3^ Shaanxi Engineering Research Center of Forage Plants of the Loess Plateau, Yulin University, Yulin, Shaanxi, China

**Keywords:** *Medicago ruthenica*, photosynthetic efficiency, yield formation, leaf morphology, sequencing

## Abstract

*Medicago ruthenica* is closely related to *Medicago sativa,* a commonly cultivated forage. Characterized by its high tolerance to environmental stress, *M. ruthenica* is a valuable genetic resource. However, low yield limits its large-scale utilization. Leaf morphology, an important agronomic trait, is closely related to forage yield and photosynthetic efficiency. In the presented study, “Correlation of Leaf Morphology and Photosynthetic Performance with Forage Yield in *Medicago ruthenica*: The Underlying Molecular Mechanisms,” comprehensive data analysis revealed a significant positive association between leaf width and leaf area with forage yield in *Medicago ruthenica* (*p* < 0.05). The specific cultivar “Mengnong No.1 (MN No.1) had a large leaf area, and its physiological parameters related to photosynthetic characteristics were superior. Anatomical examination revealed that the leaves of MN No.1 had strong palisade tissue and compact cell structure. Subsequent investigations, utilizing small RNA and transcriptome sequencing, discerned critical miRNA-target gene networks that underpin the high photosynthetic efficiency in *M. ruthenica*. A total of 63 differentially expressed miRNAs (DEMs) were identified, inclusive of several well-characterized miRNAs such as miR408, miR171, and miR398. These miRNAs were predicted to target 55 genes (mRNAs), of which 6 miRNA-target gene pairs, particularly those involving miR408and miR171, exhibited inverse expression patterns. Among the six postulated miRNA-target gene pairs, the targeted cleavage of LACCASE5 (*LAC5*) by miR408 was conclusively validated through degradome sequencing, with the cleavage site pinpointed between the 9th and 10th nucleotides from the 5′end of miR408 via the 5′-RLM-RACE assay. Therefore, it is posited that the miR408-*MrLAC5* module constitutes a central mechanism in fostering high photosynthetic efficiency in *M. ruthenica*. Moreover, these findings also provide valuable information for further study of the regulatory genes and miRNA functions of forage yield in legume forage.

## 1 Introduction

Increasing forage yield has always been a priority for the development of herbivorous animal husbandry industry. Photosynthesis has been shown to tightly affect plant yield accumulation, which can be greatly influenced by the solar radiation capture and photosynthetic efficiency (Jansson et al., 2018). Moreover, leaf shape, the important photosynthetic organ in plants, strongly affects plant architecture and yield (Murchie et al., 2009; Rowland et al., 2020). Different leaf morphology results in varied photosynthetic performance among different cultivars or the same cultivars in different environments (Ren et al., 2018). As has been evidenced previously, leaves must be wide enough for absorbing sufficient light energy, and flat and thin enough for facilitating gas exchange (CO_2_, O_2_, and H_2_O) (Tsukaya, 2006). Leaf morphogenesis is mainly influenced by genetic and environmental factors; leaves may exhibit considerable morphological diversity among species and cultivars (Tsukaya, 2018). Additionally, light conditions can also influence leaf structure; low light intensity can produce one layer of palisade tissue with a round shape, whereas under high light conditions, palisade cells present in two or three layers and polarized in the direction of leaf thickness with column shape (Tsukaya, 2005). Therefore, the optimization of leaf morphology and anatomy are important to breed high photosynthetic efficiency forages.

MicroRNAs (miRNAs) are RNAs with 19–24 nucleotide (nt) in length in eukaryotes. miRNAs play important roles in the whole process of plant growth, which is mediated by regulating the expression of target genes at the post-transcriptional level through direct cleavage or inhibition of translation (Dong et al., 2022). High-throughput sequencing is the most popular technique for identifying key traits in plants. Transcriptome and small RNA sequencing correlation analysis is a powerful approach to identify gene regulatory networks. Degradome sequencing is currently accepted as a promising method for combining high-throughput sequencing with large-scale miRNA target screening (Cakir et al., 2016; Garg et al., 2019)**.** Its main principle is that the vast majority of miRNAs in plants regulate the expression of target genes by splicing, and the cleavage sites usually occurs on the 10th or 11th nucleotide in the complementary region of miRNA and mRNA, and bioinformatics analysis is combined to screen the target genes of miRNA (Allen et al., 2004; Donovan et al., 2011). Accumulating studies have provided evidence that miRNAs participate in modulating plant morphology, yield, photoresponse, etc. (Voinnet, 2009; Zha et al., 2013b; Rogers and Chen, 2013). The conserved miR408 family has been identified in many plant species (Axtell and Bowman, 2008; Kozomara and Griffiths-Jones, 2011). The basic function of miR408 in plants has been reported. As previously reported, miR408 expression can be induced in plant by different abiotic stresses, such as copper (Cu), light, and low-temperature stress (Lu et al., 2005; Kantar et al., 2010; Trindade et al., 2010; Zhang and Li, 2013; Pan et al., 2018).

Cu is an indispensable transition metal for plants. Several plant miRNAs can be induced by Cu deficiency, and can post-transcriptionally repress the transcripts encoding Cu proteins which are hence named as Cu-miRNAs (Burkhead et al., 2009; Lu et al., 2011; Pilon, 2017). It has been shown that miR408 targets several genes encoding Cu-binding proteins; these proteins are members of laccase and phytocyanin (Pilon, 2017). Laccases (such as *LAC3*, *LAC4*, *LAC17*, and *LAC11*) are Cu-containing oxidase enzymes, which are closely implicated in lignin monomer synthesis (Ranocha et al., 2002; Zhao et al., 2013a; Schuetz et al., 2014). Plastocyanin is also a cu-binding protein, which is the reaction centre of photosystem I (PSI) and acts as an electron transport carrier in eukaryotic photosynthetic organisms (Raven et al., 1999; Joliot and Joliot, 2006). Currently, it has been proposed that miR408 can facilitate Cu delivery to plastocyanin, thereby regulating photosynthesis (Zhang et al., 2014). As has been evidenced, miR408 overexpression in model plants indeed improves photosynthetic performance (Pan et al., 2018). Additionally, multiple studies have confirmed that miR408 overexpression can increase leaf area, plant height, plant biomass, seed yield, etc. (Zhang et al., 2017; Pan et al., 2018; Song et al., 2018).

*Medicago ruthenica* (L.), as a perennial legume forage (Small and Marcel, 1989), is closely related to alfalfa (*Medicago sativa*), which is an excellent germplasm material for cross breeding of *Medicago* genus (Guan et al., 2009; Yang et al., 2011). *M. ruthenica* is highly adaptable to drought, low temperature, high temperature, saline-alkali and other harsh environment, so it is considered as a valuable forage for both artificial and natural grasslands (Campbell et al., 1997; Campbell et al., 1999). However, the forage yield and seed yield of *M. ruthenica* is low, which limits its large-scale popularization and application. Therefore, high-yield breeding has always been an important direction of *M. ruthenica* breeding (Zhang et al., 2018). It has been shown that plant architecture is fundamentally important to plant growth and productivity (Wang et al., 2018; Zhang and Shi, 2020). The plant architecture of *M. ruthenica* varies significantly, and its germplasm resources differ greatly in growth habit, leaf shape, plant height, and seed size (Campbell et al., 1999; Zhang et al., 2022b). Recently published genome information provides a powerful reference for investigating the mechanisms of yield-related agronomic characteristics in *M. ruthenica* (Wang et al., 2021).

In this study, the forage yield, photosynthetic characteristics, and yield-related traits of three *M. ruthenica* cultivars were compared. It was found that there was a significant positive correlation between leaf morphological characteristics and forage yield in *M. ruthenica*. Namely, the cultivar with wide leaves and large leaf area showed the highest photosynthetic utilization efficiency and high yield. To reveal the mechanisms of high photosynthetic efficiency in *M. ruthenica* leaves, we performed a multiomics analysis to identify the potential regulation of miRNA-target pairs in *M. ruthenica* leaf morphogenesis. The results of the present work explored the molecular regulatory network for the high photosynthetic efficiency of *M. ruthenica*. Moreover, this study provided important guiding significance for the bio-design of high-yield *M. ruthenica*.

## 2 Results

### 2.1 Leaf morphological and anatomical characteristics of three *M. ruthenica* cultivars

#### 2.1.1 Leaf morphological characteristics analysis

**FIGURE 1:**
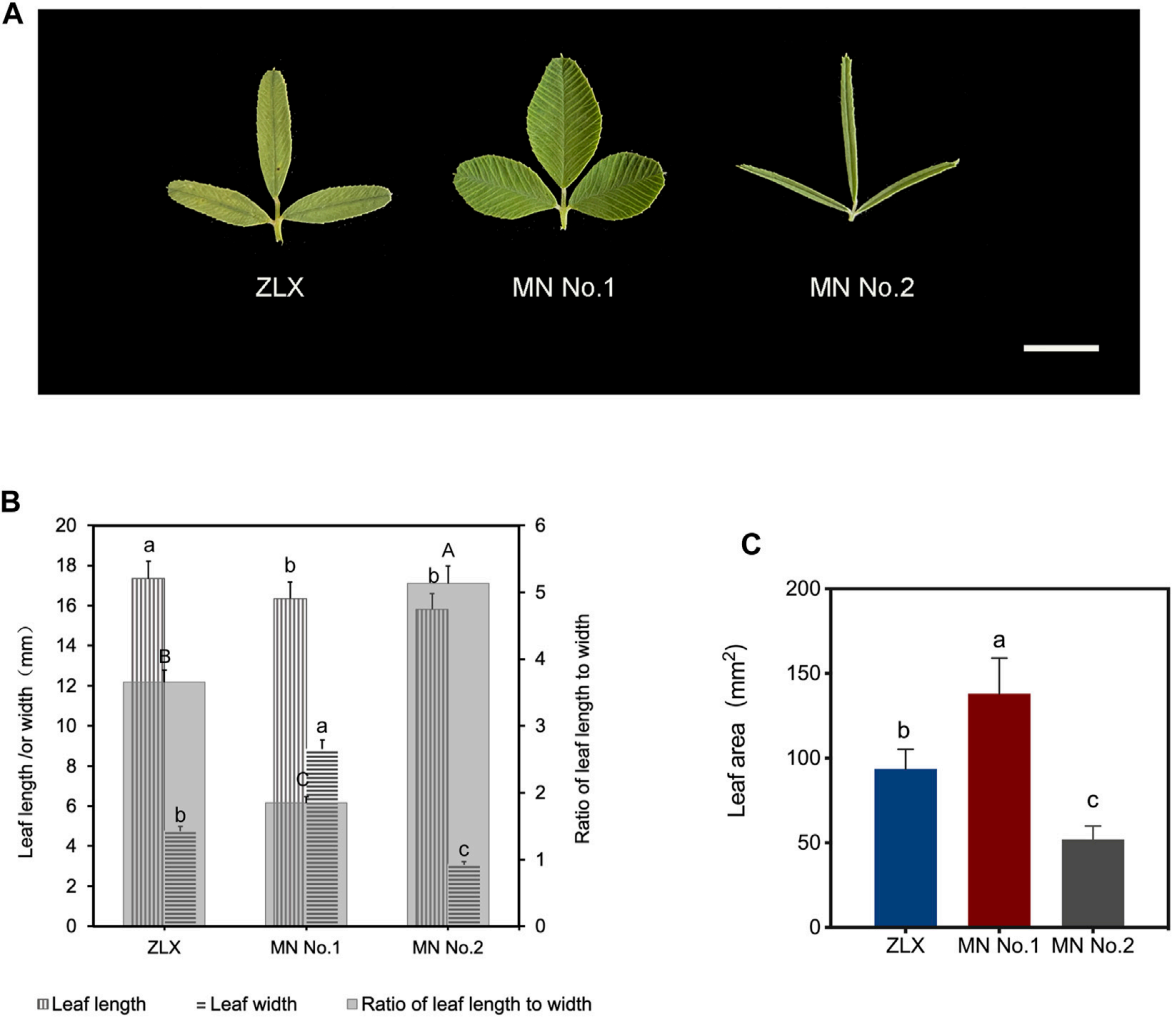
Leaf morphology of three *M. ruthenica* cultivars. **(A)** Leaves of three *M. ruthenica* cultivars at the early flowering stage. Bar = 1 cm. **(B)** Comparison of the leaf length, leaf width, and the ratio of leaf length to width among three *M. ruthenica* cultivars. Different lowercase letters indicated significant differences in the leaf length (or width) of the three cultivars, and different uppercase letters indicated significant differences in the ratio of leaf length to width of the three cultivars (*p* < 0.05). **(C)** Comparison of leaf area among three three *M. ruthenica* cultivars. In **(B, C)**, values are means ± S.D. of twenty biological replicates.

As shown by the varietal yield test results of three *M. ruthenica* cultivars (ZLX, MN No.1, and MN No.2), MN No.1 was outstanding in high yield and its 3-year average hay yield increased by 12.56%–19.20% compared with the other two cultivars (*p* < 0.05, Supplementary Table S1). Correlation analysis of leaf morphological characteristics and yield-related traits showed that leaf width was highly significantly correlated with hay yield (*p* < 0.01), and leaf area was significantly correlated with absolute plant height and hay yield (*p* < 0.05, Supplementary Table S2). The leaf morphology of three *M. ruthenica* cultivars was notably different (Figure 1A). Specifically, the length-width ratio of MN No.1 had a length-to-width ratio of 1.85, showing a broad oval shape; ZLX was oval with a ratio of 3.65; MN No.2 was in the shape of a long strip with a length-to-width ratio of 5.13 (Figure 1B). The leaf area of MN No.1 was 32.12% and 62.31% larger than that of ZLX and MN No.2, respectively (*p* < 0.05, Figure 1C).

#### 2.1.2 Leaf morphological characteristics analysis

**FIGURE 2:**
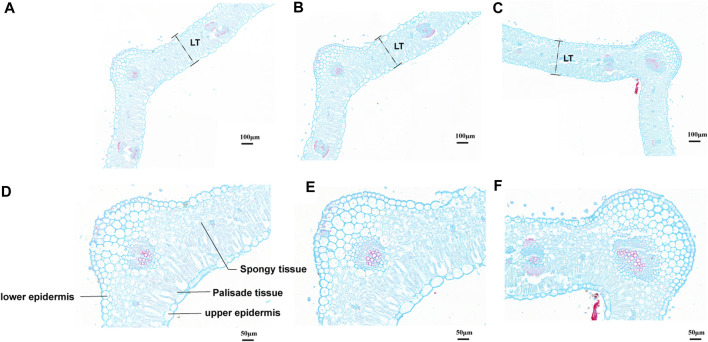
Leaf cross-section of three *M. ruthenica* cultivars stained with Safranin O/Fast Green. **(A, D)**, ZLX. **(B, E)**, MN No.1. **(C, F)**, MN No.2. **(A–C)**, at ×15.0 magnifications, bar = 100 μm. **(D**–**F),** at ×35.0 magnifications, bar = 50 μm. The leaf thickness was measured according to the " LT ″ of **(A–C)** labels. The schematic diagram of the leaf structure is marked in **(D–F)** are the same.

According to the leaf anatomic staining results, *M. ruthenica* showed typical dorsoventral leaves; the mesophyll was divided into palisade tissue and spongy tissue (Figure 2). The palisade tissue thickness of MN No.1 103.85 μm, which was significantly higher than that of ZLX (96.46 μm) and MN No.2 (89.15 μm), while its spongy tissue thickness (58.76 μm) was notably lower than the other two cultivars (*p* < 0.05, Table 1). Therefore, MN No.1 had the largest thickness ratio of palisade tissue to spongy tissue (1.76), indicating that it had developed palisade tissue. Additionally, among these three *M. ruthenica* cultivars, the highest cell tense ratio (CTR) of MN No.1 leaves was the highest (42.43), and the CTR of ZLX and MN No.2 leaves were 40.83 and 39.54, respectively, indicating that the cell structure of MN No.1 leaves was the most compact, while that of ZLX and MN No.2 was relatively loose. Moreover, MN No.1 leaves showed a dramatically higher thickness of the upper epidermis (16.52 μm) and lower epidermis (14.93 μm) than ZLX and MN No.2 (*p* < 0.05, Table 1).

**TABLE 1:** Statistics of anatomic characteristics of leaf cross-section of three *M.ruthenica* cultivars.

Leaf structure	Cultivar		
	ZLX	MN No.1	MN No.2
Upper epidermis thickness/μm	13.43 ± 0.32b	16.52 ± 0.53a	14.57 ± 0.40b
Lower epidermis thickness/μm	13.16 ± 0.51ab	14.93 ± 0.58a	14.06 ± 0.32b
Leaf thickness/μm	381.72 ± 6.66ab	418.71 ± 27.8a	350.34 ± 4.88b
Palisade tissue thickness/μm	96.46 ± 1.10ab	103.85 ± 3.52a	89.15 ± 4.28b
Spongy tissue thickness/μm	78.32 ± 1.94a	58.76 ± 2.50c	74.43 ± 2.75a
Thickness ratio of palisade tissue to spongy tissue	1.23 ± 0.03b	1.76 ± 0.07a	1.20 ± 0.05b
Cell tense ratio (CTR)	40.83 ± 0.53ab	42.43 ± 1.32a	39.54 ± 0.69b
Spongy ratio (SR)	37.37 ± 0.80ab	35.78 ± 0.74b	38.48 ± 0.85a

Note: Completely different lowercase letters on the same line indicate significant differences (*p* < 0.05). CTR= (palisade tissue thickness/leaf thickness) ×100%, SR = (sponge tissue thickness/leaf thickness) ×100%.

### 2.2 Leaf photosynthetic characteristics of three *M. ruthenica* cultivars

#### 2.2.1 Photosynthetic pigment content in leaves

Chlorophyll (Chl) plays an important role in light absorption during photosynthesis. The Chl concentration of three *M. ruthenica* cultivars with different leaf morphology was compared (Table 2). MN No.1 had the highest content of chlorophyll a (Chl a), chlorophyll b (Chl b), total chlorophyll (Chl t), and carotenoids (Car), followed by ZLX and then MN No.2. Moreover, the photosynthetic pigment content indexes of MN No.1 and ZLX were significantly higher than those of MN No.2 by 11.63%–36.17% (*p* < 0.05) (Table 2). Additionally, the ratio of chlorophyll a to chlorophyll b (Chl a/b) of MN No.1 and ZLX was 3.92 and 4.09, respectively, which were notably higher than that of MN No.2 (*p* < 0.05) (Table 2).

**TABLE 2:** Photosynthetic pigment content in leaves of three *M.ruthenica* cultivars.

Cultivar	Chl a (mg·g^−1^)	Chl b (mg·g^−1^)	Chl t (mg·g^−1^)	Chl a/b	Car (mg·g^−1^)
ZLX	1.76 ± 0.03a	0.43 ± 0.02a	2.19 ± 0.17a	4.09 ± 0.10a	0.37 ± 0.11ab
MN No.1	1.88 ± 0.06a	0.48 ± 0.03a	2.36 ± 0.16a	3.92 ± 0.07a	0.42 ± 0.02a
MN No.2	1.20 ± 0.02b	0.38 ± 0.03b	1.58 ± 0.08b	3.16 ± 0.04b	0.27 ± 0.02b

Note: Completely different lowercase letters on the same column indicate significant differences (*p* < 0.05).

#### 2.2.2 Photosynthetic response parameters

**FIGURE 3:**
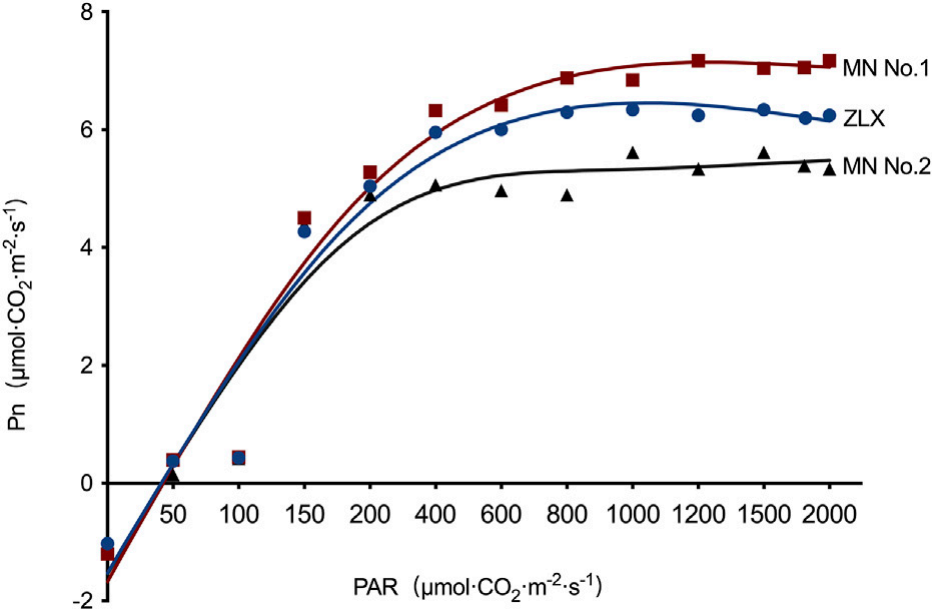
The effect of light intensity on the Pn of three *M.ruthenica* cultivars. Pn, net photosynthetic rate. PAR, photosynthetically active radiation.

Subsequently, the effect of light intensity on the net photosynthetic rate (Pn) of three *M. ruthenica* cultivars was explored. Under low light intensity (PAR <200 μmol·CO_2_·m^-2^·s^-1^), the Pn of three *M. ruthenica* cultivars increased linearly, but the increasing amplitude was gradually decreased with the increase of light intensity. There were significant differences in Pn among these three cultivars (Figure 3). Based on the leaf light response curve, photosynthetic characteristic parameters were calculated (Table 3). The results showed that maximum Pn (P_max_), apparent quantum efficiency (AQE) and light saturation point (LSP) of MN No.1 were 7.29 μmol·m-^2^·s^-1^, 0.0453 μmol·m-^2^·s^-1^, and 224.30 μmol·m-^2^·s^-1^, respectively. Compared with ZLX and MN No.2, it was significantly increased by 7.04%–11.62%, 23.84%–45.69% and 15.91%-25.51, respectively (*p* < 0.05). However, the dark respiration rate (R_d_, 1.06 μmol·m-^2^·s^-1^) and light compensation point (LCP, 21.56 μmol·m-^2^·s^-1^) of MN No.1 were significantly lower than those of other two cultivars (*p* < 0.05) (Table 3). In conclusion, MN No.1 exhibited the strongest light energy utilization efficiency.

**TABLE 3:** Photosynthetic response parameters of three *M.ruthenica* cultivars.

Cultivar	LSP (μmol·m-^2^·s^-1^)	LCP (μmol·m-^2^·s^-1^)	AQE(μmol·m-^2^·s^-1^)	R_day_(μmol·m-^2^·s^-1^)	P_max_(μmol·m-2·s-1)
ZLX	208.50 ± 10.67b	26.64 ± 0.22a	0.0345 ± 0.0002b	1.24 ± 0.020b	6.13 ± 0.19b
MN No.1	224.30 ± 2.33a	21.56 ± 0.31b	0.0453 ± 0.0010a	1.06 ± 0.001c	7.29 ± 0.21a
MN No.2	198.23 ± 2.61b	26.88 ± 0.36a	0.0246 ± 0.0003c	1.43 ± 0.010a	5.43 ± 0.07c

Note: Different lowercase letters on the same column indicate significant differences (*p* < 0.05).

### 2.3 Small RNA sequencing and identification of differentially expressed miRNAs in three *M. ruthenica* cultivars

To identify miRNAs involved in the morphology of *M. ruthenica* leaves, we performed small RNA sequencing of nine leaf samples (three leaves sample for each cultivar). An overview of reads from raw data to cleaned sequences is listed in Supplementary Table S3. A total of 57,594,597, 108,949,242, and 83,342,015 raw reads were generated from MN No.1, MN No.2, and ZLX, respectively. After deleting low-quality sequences, the obtained sequences (18–25 nt in length) from three cultivars were 35,743,959 (MN No.1), 55,059,742 (MN No.2) and 49,372,600 (ZLX), respectively (Supplementary Table S4).

**FIGURE 4:**
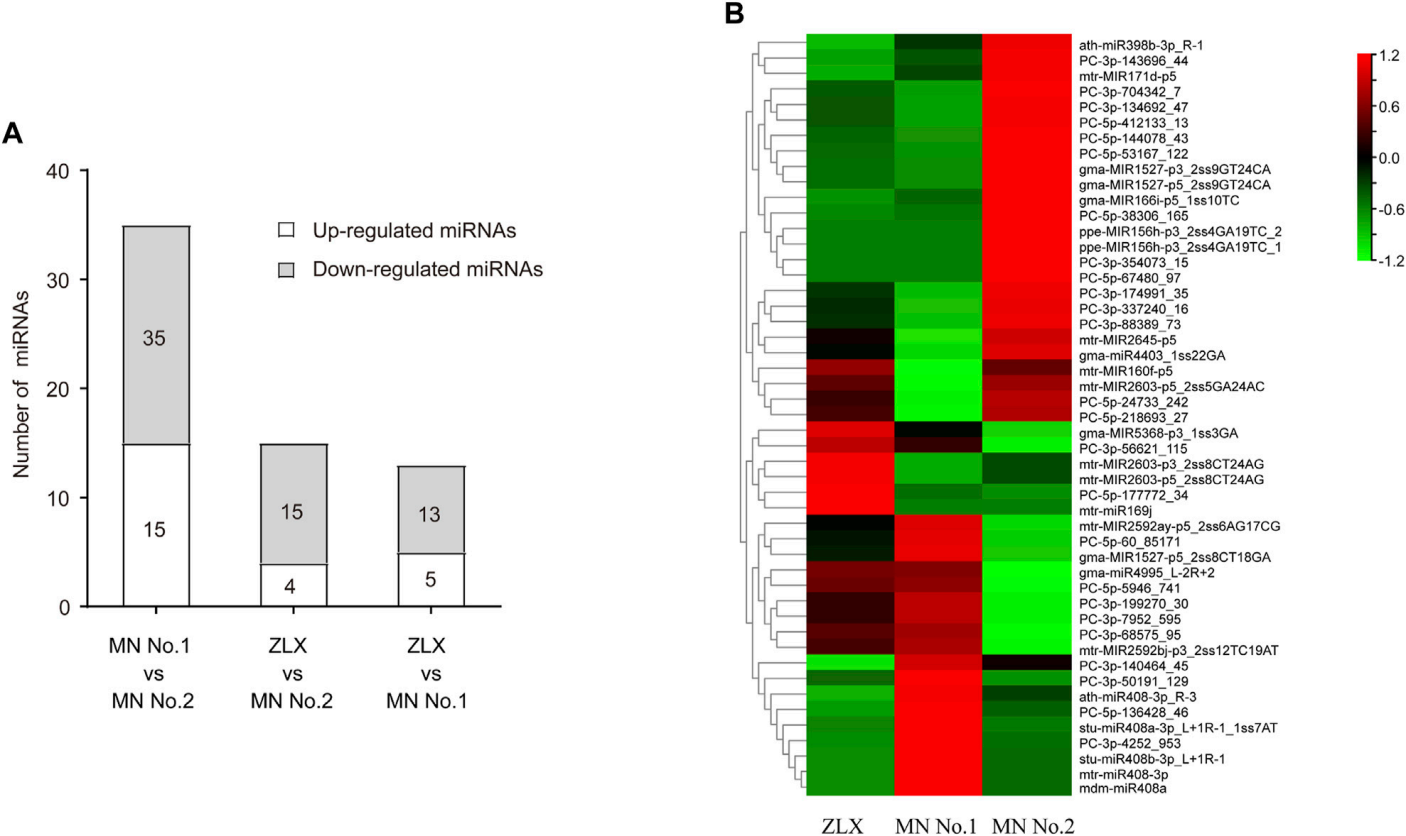
Differentially expressed miRNAs (DEMs) in three *M. ruthenica* cultivars. **(A)** The number of DEMs among the three cultivars. **(B)** DEMs in three *M. ruthenica* cultivars by miRNAs hierarchical clustering. Red indicates high-levels of miRNAs and green indicates low-level miRNAs. The original expression values of the miRNAs were normalized using Z-score normalization. The absolute signal intensity ranges from −1.5 to +1.5, with corresponding color changes from green to red.

The expression levels of miRNAs in three cultivars were analyzed and compared. A total of 63 miRNAs with differential expression level was identified (*p* < 0.01, Supplementary Table S5). The numbers of DEMs among the three cultivars was identified. There were 35, 15, and 13 DEMs in the MN No.1 vs. MN No.2, ZLX vs. MN No.2, and ZLX vs. MN No.1 comparisons, respectively (Figure 4A). As shown by the heatmap of DEMs the identified DEMs included 24 known miRNAs (such as miR398b, miR171d, miR156h, miR160f, miR169j, and miR408), and 39 novel miRNAs. Members of the same miRNA family showed consistent expression patterns. For example, five miR408s were significantly upregulated in the cultivar MN No.1, while their expression levels were downregulated in both ZLX and MN No.2 (Figure 4B).

### 2.4 Annotation and enrichment analysis of the target genes for DEMs

**FIGURE 5:**
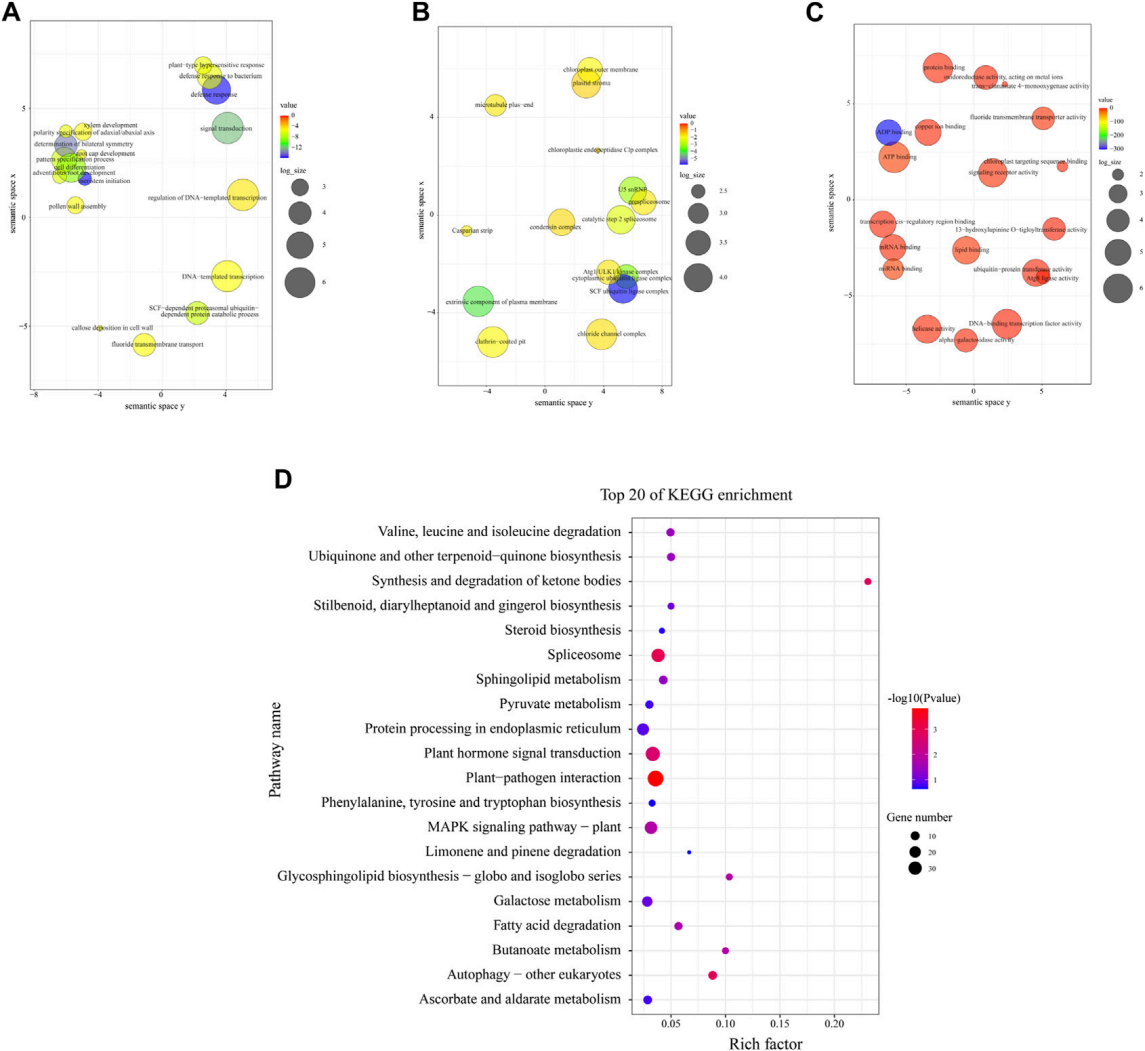
Gene ontology (GO) and Kyoto Encyclopedia of Genes and Genomes (KEGG) functional enrichment analyses of the identified target genes. **(A–C)** GO enrichment analysis for the targets of DEMs. **(D)** KEGG enrichment analysis for the targets of DEMs.

Transcriptome libraries were constructed, and the transcriptome sequencing (RNA-seq) details of 9 samples in *M. ruthenica* are shown in Supplementary Table S6. According to the results, a total of 5047 and 457 genes were annotated with enriched GO terms and KEGG pathways, respectively. Additionally, 816 target genes for DEMs were obtained from three libraries; 689 target genes were annotated into three main GO categories: biological process, cellular component, and molecular function (Figures 5A–C). Under biological processes, leaf morphogenesis-associated terms were identified, such as meristem initiation (GO:0010014), signal transduction (GO:0007165), DNA-templated transcription (GO:0006351), cell differentiation (GO:0030154), polarity specification of adaxial/abaxial axis (GO:0009944), xylem development (GO:0010089), and callose deposition in cell wall (GO:0052543) (Figure 5A). Under the cellular component, SCF ubiquitin ligase complex (GO:0019005), extrinsic component of plasma membrane (GO:0019897), cytoplasmic ubiquitin ligase complex (GO:0000153) and U5 snRNP (GO:0005682) were the most significantly enriched terms (*p* < 0.001, Figure 5B). Under molecular function, all terms were notably enriched, including ADP binding (GO:0043531), ATP binding (GO:0005524), miRNA binding (GO:0035198), signaling receptor activity (GO:0038023), copper ion binding (GO:0005507), etc. (Figure 5C).

The top 20 most enriched KEGG pathways are shown in Figure 5D. It was found that plant-pathogen interaction (ko04626), spliceosome (ko03040), autophagy-other eukaryotes (ko04136), synthesis and degradation of ketone bodies (ko00072), and plant hormone signal transduction (ko04075) were the remarkably enriched pathways (*p* < 0.01)**,** which indicated that these pathway might interplay in leaves morphogenesis, leaves anatomy development or leaves physiological processes of *M. ruthenica*.

### 2.5 Correlation analysis of the expression profiles of the miRNAs and their target genes

For investigating the genes regulating leaf morphology in *M. ruthenica*, DEGs among the three cultivars were analyzed. As shown by RNA-seq results, 1540, 1956 and 3014 DEGs were identified among three comparison groups (ZLX vs MN No.1, ZLX vs MN No.2, and MN No.1 vs MN No.2), respectively (Supplementary Figure S1; Supplementary Table S7).

**FIGURE 6:**
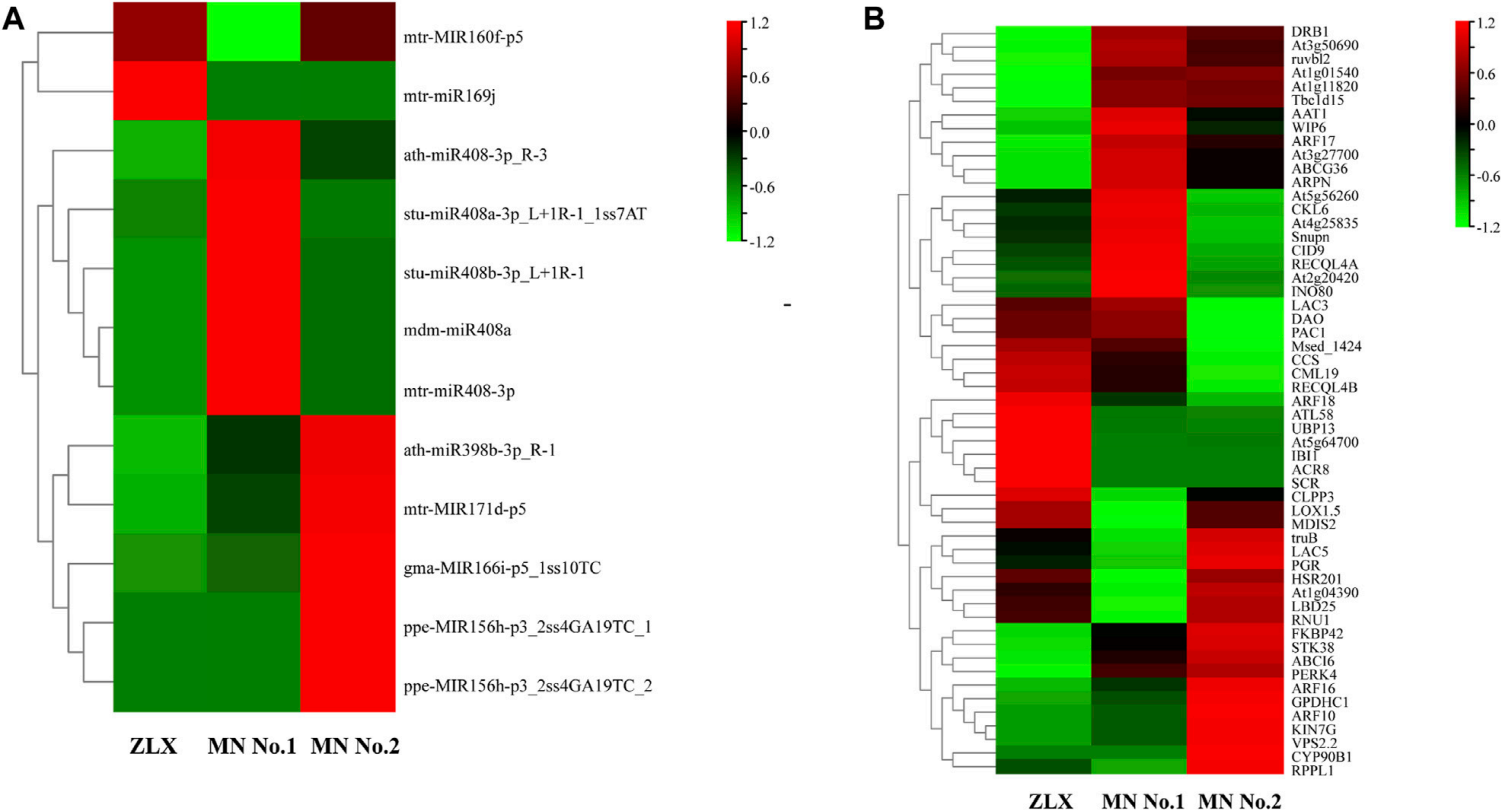
The expression level of the known DEMs **(A)** and their target genes **(B)** in three *M. ruthenica* cultivars. The original expression values of miRNAs and their target genes were normalized by Z-score normalization.

In a survey, the expression level of miRNAs in miR160, miR169, miR408, miR398, miR171, miR166, and miR156 families was significantly different among three *M. ruthenica* cultivars. The expression levels of miR160 and miR169 in MN No.1 were significantly downregulated (MN No.1 vs. ZLX). And miR398, miR171, miR166, and miR156 were significantly downregulated in MN No. 1 (or ZLX), which was more than 2.5 times lower than that in MN No.2. However, the expression level of miR408 in MN No.1 was 3-4-fold higher than that in ZLX and MN No.2 (*p* < 0.01, Figure 6A). Subsequently, a total of 55 target genes corresponding to these key known DEMs were identified (Figure 6B). A total of 8 pairs of miRNA-known target genes were significantly different among these three groups, in which 6 pairs were negatively correlated (Table 4). For example, ath-miR408-3p was significantly upregulated in the cultivar MN No.1, while its target gene *HSR201*, *PGR*, and *LAC5* were notably downregulated in MN No.1. mtr-miR171d was downregulated, while its target gene *SNUPN* was upregulated in MN No.1 (MN No.1 vs MN No.2).

**TABLE 4:** The miRNA-known target gene pairs showed significantly differentially expressed in the three comparison groups.

Comparison	miRNA name	miRNA expression level	Target name	Target expression level
MN No.1 vs MN No.2	ath-miR408-3p	upregulation	*HSR201*	downregulation
	ath-miR408-3p	upregulation	*LAC3*	upregulation
	ath-miR408-3p	upregulation	*PGR*	downregulation
	ath-miR408-3p	upregulation	*LAC5*	downregulation
	mtr- miR171d	downregulation	*Snupn*	upregulation
ZLX vs MN No.1	ath-miR408-3p	downregulation	*HSR201*	downregulation
	ath-miR408-3p	downregulation	*ACR8*	upregulation
ZLX vs MN No.2	ath-miR398b-3p_R-1	downregulation	*At5g56260*	downregulation
	mtr-miR171d	downregulation	*At5g64700*	upregulation

**FIGURE 7:**
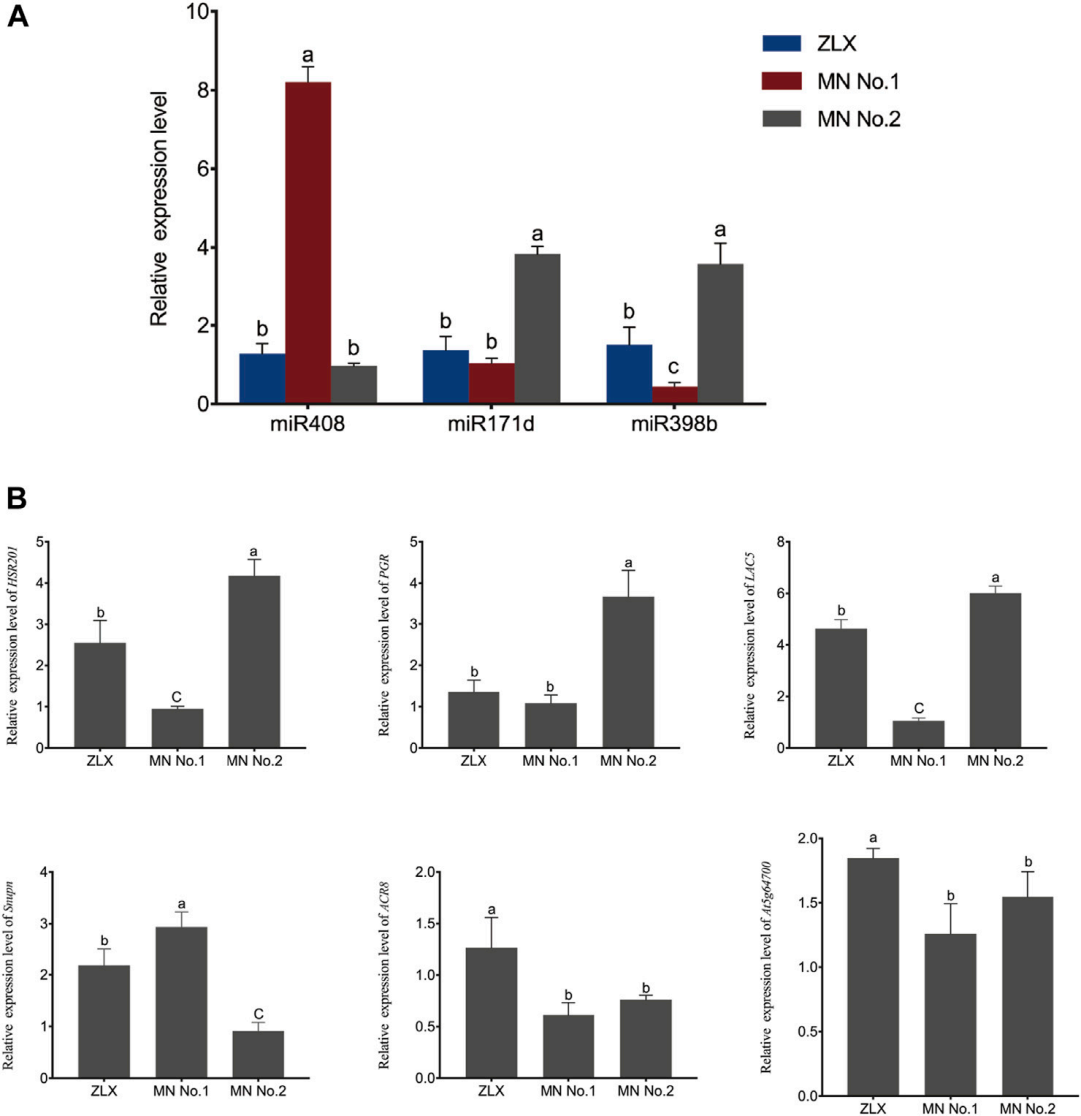
Relative expression level of miRNAs and their targets in three *M. ruthenica* cultivars. MrU6 SnRNA and *MrACTIN* served as the internal control for miRNAs and mRNAs, respectively. In **(A, B)**, values are means ± S.D. of three technical replicates from three biological replicates. Different lowercase letters indicate significant differences (*p* < 0.01).

Furthermore, the expression of these miRNA–target pairs was verified using qRT-PCR. The expression trends of 3 miRNAs (miR408, miR171d, and miR398b) and 6 target genes (*HSR201*, *PGR*, *LAC5*, *SNUPN*, *ACR8*, and *At5g64700*) were similar to those obtained by deep sequencing (Figure 7). The negative correlation of the expression level of these miRNA-target pairs was confirmed. Namely, miR408 family members were notably upregulated in the cultivar MN No.1 (*p* < 0.01, Figure 7A), while their target genes *HSR201*, *PGR*, *LAC5,* and *ACR8* showed significant opposite expression patterns (*p* < 0.01, Figure 7B). miR171d was significantly upregulated in the cultivar MN No.2 (*p* < 0.01, Figure 7A), while its target genes *SNUPN* and At5g64700 were remarkably downregulated (*p* < 0.01, Figure 7B).

### 2.6 Identification of miRNA targets via degradome sequencing in *M. ruthenica*

**FIGURE 8:**
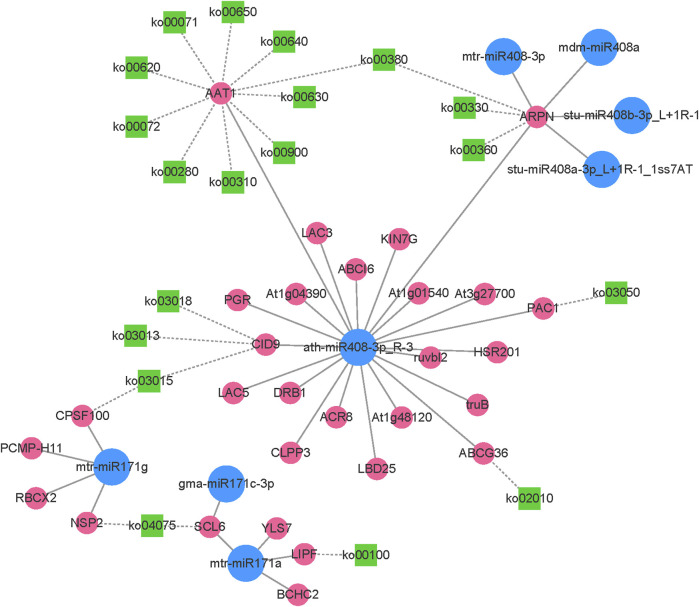
The network plot of the miR408, miR171 and their target genes.

After integrating transcriptome and small RNA sequencing data, the expression network of miR408, miR171, and their target genes was constructed (Figure 8). In the network, 5 miR408 family members and 3 miR171 family members were identified. The network plot for miR408 and its target genes was associated with 17 known KEGG pathways (Supplementary Table S8), for instance, ko00620 (Pyruvate metabolism), ko00380 (Tryptophan metabolism), ko00330 (Arginine and proline metabolism), ko02010 (ABC transporters), and ko03015 (mRNA surveillance pathway) etc. This suggests that miR408 plays an important role in the regulation of multiple metabolic pathways. miR171 and its target genes involved 3 known KEGG pathways (Supplementary Table S8), including ko03015, ko04075 (Plant hormone signal transduction), and ko00100 (Steroid biosynthesis). The ko03015 was found involved both in ath-miR408-3p_R-3 and mtr-miR171g.

**FIGURE 9:**
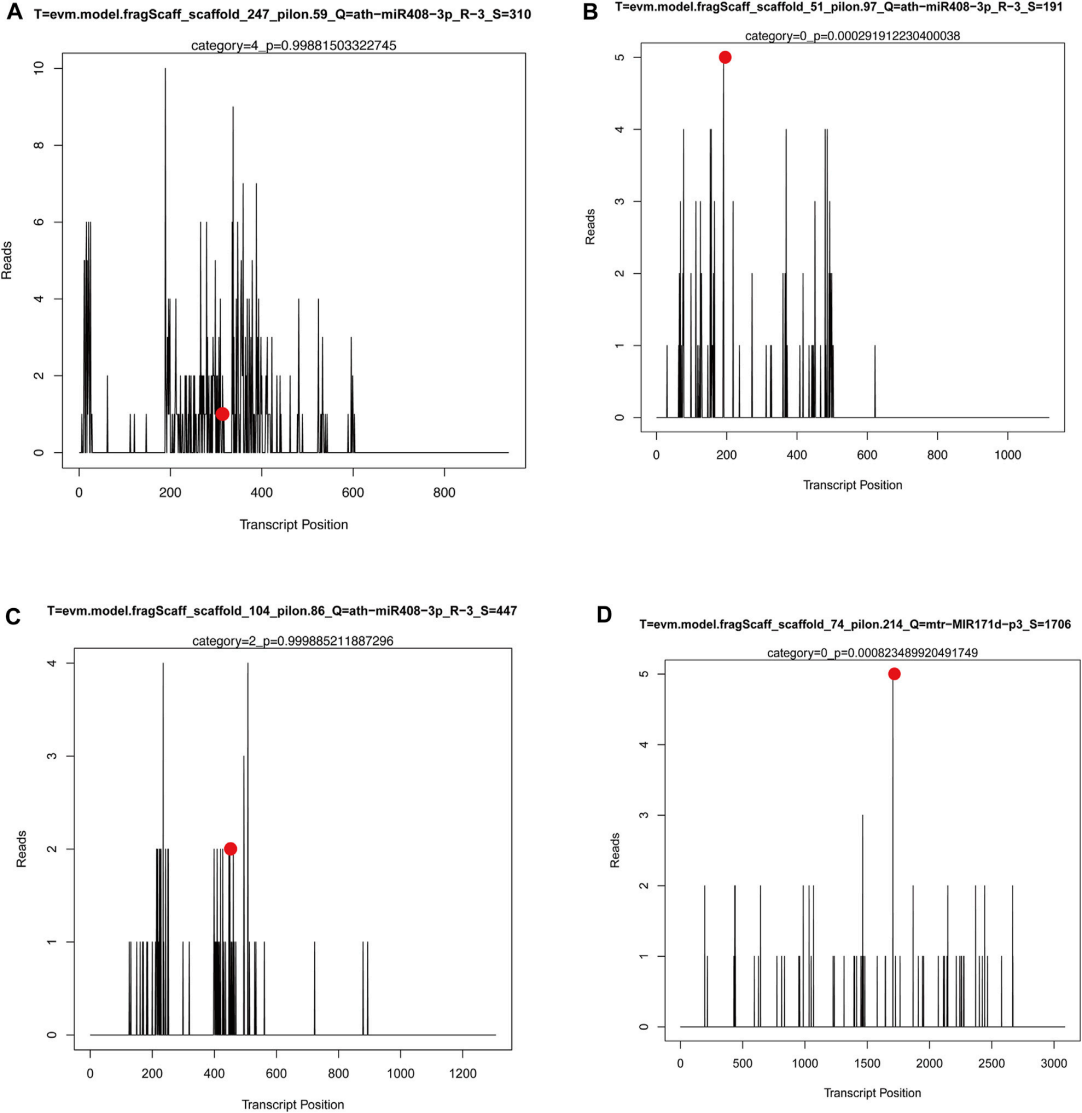
The T-plots for the target genes of miRNAs by degradome sequencing. *X*-axis represents the site position of target transcript. *Y*-axis represents the normal abundance of raw tags. Red circle reresents the cleavage site.

However, the authenticity of miRNAs cleaving target genes needs to be further verified. In this study, the authenticity of miRNAs and their predicted target genes in three groups of *M. ruthenica* was further verified using degradome sequencing. The miRNAs-target genes association analysis based on degradome sequencing has been summarized in Supplementary Table S9. After transcriptome and small RNA sequencing analysis, the miRNA-target gene pairs were obtained, mainly involving miR408, miR171, and their target genes. Several key target plots (T-plots) of miR408, miR171 and their target genes validated by degradome sequencing are shown in Figure 9. It was observed that ath−miR408−3p_R−3 could cleave the target gene *LAC5* (evm.model.fragScaff_scaffold_247_pilon.59) through a specific site (Figure 9A), however, the other predicted target genes were not matched in the degradome sequencing. Interestingly, degradome sequencing results revealed some more plausible miRNA-target gene pairs, such as miR408-targeted cleavage *ARPN* (evm.model.fragScaff_scaffold_51_pilon.97) (Figure 9B) and *NAP-1* (evm.model.fragScaff_scaffold_104_pilon.86) (Figure 9C), and miR171-targeted cleavage *SCL6* (evm.model.fragScaff_scaffold_74_pilon.214) (Figure 9D). These findings may provide clues to elucidate the mechanism of other morphological or physiological processes in *M. rutheni*ca.

### 2.7 Experimental validation of key miRNA target in *M. ruthenica*

**FIGURE 10:**
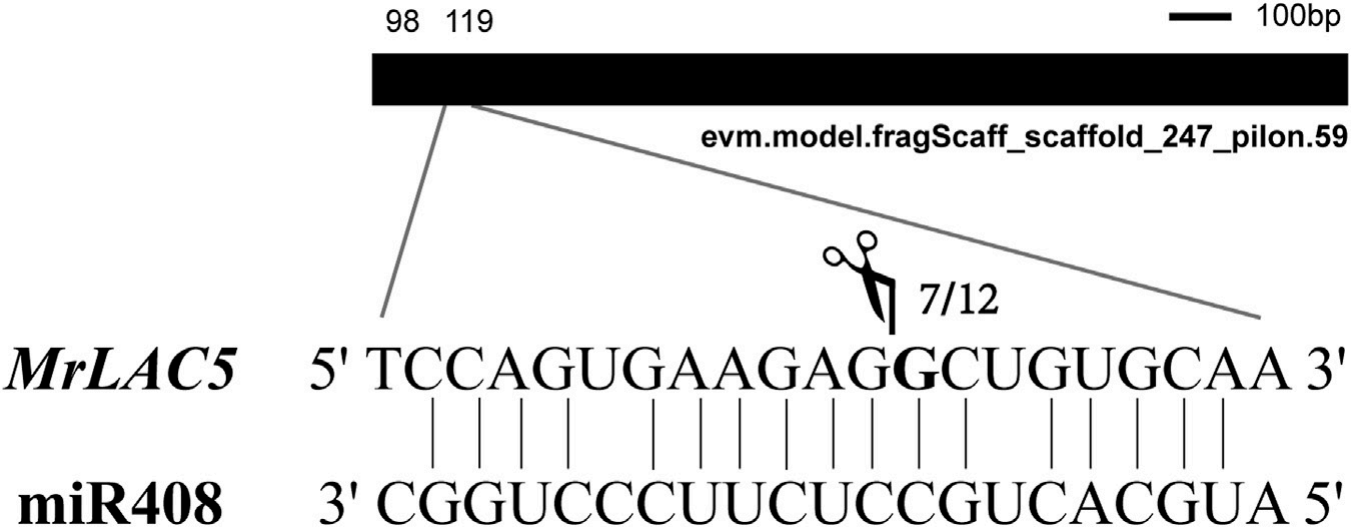
Experimental validation of miR408-targeted cleavage *MrLAC5*. The mRNA (*MrLAC5*) cleavage site were determined by 5′-RLM-RACE. Heavy black lines represent unigenes. miR408 complementary sites with the nucleotide positions of *MrLAC5* are indicated. Scissors vertical line represents the 5′termini of miR408-targeted cleavage products. Numbers show the frequency of clones.

To further confirm whether miR408, which is differently expressed among the three cultivars, can cleave the predicted target (*MrLAC5*), we isolated RNAs from the leaves of MN No.1, MN No.2, and ZLX of *M. ruthenica* and mixed them in equal amounts to perform a 5′-RLM-RACE experiment to verify the cleavage site. The results showed that the 5‘end of the cleaved fragment was the same site as *MrLAC5* in seven of the twelve clones randomly selected. The cleavage site is located between the 9th and 10th nucleotides of miR408 (Figure 10). The above results confirmed that *MrLAC5* is a target of miR408 in *M. rutheni*ca, and its expression level depends on the miR408-directed cleavage patterns of the miR408.

## 3 Discussion

It has been widely accepted that enhancing photosynthetic efficiency is critical for advancing forage crop yield (Nelson et al., 1975; Zhu et al., 2010; Wu et al., 2019). Plant architecture and leaf morphology can influence light intensity, thus affecting the photosynthetic ability of plants (Mathan et al., 2016). These characteristics can be considered to play a role in dictating forage performance and yield; optimizing these developmental characteristics is essential for high-yield forage breeding. Our results showed a significant positive correlation between leaf morphology and hay yield in *M. ruthenica* (*p* < 0.01). The photosynthetic indexes of 3 *M. ruthenica* cultivars were compared. The broad-leaved cultivar (MN No.1) had the highest photosynthetic pigment content and Pn. Namely, the *M. ruthenica* cultivars with high photosynthetic efficiency had a large leaf area. Consistently, Tsukaya (Tsukaya, 2006) has also reported that leaf morphology is the key to affecting photosynthesis; leaves must be as wide as possible for absorbing sufficient light energy. Moreover, accumulating studies have provided evidence for the relationship between leaf anatomical structure and photosynthetic characteristics in many species (Chatelet et al., 2013; Xiong and Flexas, 2021; Zhang et al., 2022a). In this study, the leaves of three *M. ruthenica* cultivars were observed by paraffin sectioning to further clarify the relationship between leaf anatomical structure and high photosynthetic efficiency. The results showed that cultivar MN No.1 with high photosynthetic efficiency exhibited a greater leaf thickness than the other two cultivars. Consistent findings have also been reported by Mcclendon (Mcclendon, 1962) and Nikolopoulos et al. (Nikolopoulos et al., 2002). Thicker leaves have relatively large mesophyll cells (Tsuge et al., 1996; Villar et al., 2013), and mesophyll cells contain a large number of chloroplasts that functions as the main sites for photosynthesis in plants. The increase in mesophyll cell density leads to an enlarged leaf area, which in turn affects the photosynthetic capacity of leaves (M. Weraduwage et al., 2016). In this study, the mesophyll cells of *M. ruthenica* differentiated into palisade tissues and spongy tissues; the ratio of palisade tissues to spongy tissues was different among the three *M. ruthenica* cultivars. Consequently, MN No.1 with strong photosynthetic capacity has developed palisade tissues and high cell tightness.

Indeed, there remain many challenges in high-yield *M. ruthenica* breeding, including photosynthetic efficiency and molecular mechanisms controlling leaf morphology. It is well-accepted that miRNAs are essential regulators of gene expression and are involved in multiple aspects of plant architecture. The expression of miR160, miR169, miR398, miR166, miR156 was significantly different among the three *M. ruthenica* cultivars and they were shown to be involved in several plant physiological processes. For example, the miR160 family members target the ARFs genes that are involved in the auxin signal transduction pathway, which is critical for plant development and stress-induced responses (Li et al., 2016; Hao et al., 2022). miR169 and its key target NFYA genes participate in plant physiological and biochemical activities through an abscisic acid-mediated hormone signaling pathway (Sorin et al., 2014; Li et al., 2017). miR398 has been proposed to be directly involved in the regulatory network of plant stress, which regulates plant responses to drought stress, salt stress, oxidative stress, copper, and bacterial infection, etc. (Zhu et al., 2011). Knockdown of miR166 can improve the drought resistance of plants, which can also lead to morphological changes, such as plant dwarfing, leaf curling, and xylem diameter reduction (Li et al., 2020; Zhao et al., 2022). The regulatory network regarding the miR156-SPL module is highly conserved in different plant species, which plays important roles in regulating plant stress resistance and production performance (Wang and Wang, 2015). The expression of the above miRNAs was significantly different among the three *M. ruthenica* cultivars. Specifically, miR160f and miR169 were highly expressed in the cultivar ZLX, however, they were poorly expressed in MN No.1. miR398b, miR166i and miR156h showed high expression abundance in the cultivar MN No.2 (Figure 6A). It is worth mentioning that the predicted target genes of these miRNAs showed no significant expression difference among the three cultivars, which suggested that these target genes may also respond to other metabolic processes in the growth and development of *M. ruthenica*.

Plant miRNAs are known to negatively regulate the expression of target genes at the post-transcriptional level through cleavage or/and translational inhibition (Voinnet, 2009; Strzyz, 2021; Dong et al., 2022). In this study, 66 miRNA-target gene pairs with negative regulation were identified. miR171d showed a high expression abundance in the cultivar MN No.2, which was predicted to negatively regulate the expression of its target genes *SNUPN* (evm.model.fragScaff_scaffold_93_pilon.67) and *At5g64700* (evm.model.fragScaff_scaffold_65_pilon.357). It has been shown that miR171 regulates the SCL transcription factor family members to play important roles in plant development, stress response, and photochrome signaling, etc. (Wang et al., 2010; Zhu et al., 2015). Software prediction and degradome sequencing identified *SCL6* (evm.model.fragScaff_scaffold_74_pilon.214) as a target gene of mtr-miR171d-p3; but *SCL6* expression showed no significant expression difference among the three cultivars (Supplementary Figure S2); these findings suggested that the miR171d-*SCL* pair was not the regulator of the leaf morphogenesis of *M. ruthenica*. miR171d-*SNUPN* and miR171d-*At5g64700* pairs in *M. ruthenica* also provided a reference for the module analysis of plant architecture formation of legume forage. The other four pairs are target genes (*LAC5*, *PGR*, *HSR201*, and *ACR8*) for miR408.

miR408 is a typical multifunctional miRNA that has been identified in many plant species (Gao et al., 2022). A previous study has shown that high-level miR408 accumulation in plants can improve photosynthesis through enhancing photosynthetic efficiency and CO_2_ fixation capacity (Pan et al., 2018). As miR408 hyper-accumulation can improve the overall photosynthetic performance, its potential of increasing the yield has also been verified in several model plants (Zhang et al., 2017; Pan et al., 2018; Song et al., 2018). In the present study, miR408 was highly expressed in the cultivar MN No.1 with high photosynthetic efficiency, with its expression approximately 8-fold higher in MN No.1 than that in MN No.2 and ZLX (Figure 7A).

The results of degradation sequencing and psRobot prediction indicated that the targets genes of miR408 could encode Cu proteins, including the laccase genes *LAC3* and *LAC5*. *MrLAC5* is a target gene negatively regulated by miR408, whereas *LAC3* expression was consistent with the miR408 expression trend. We also demonstrated that miR408 targeted the cleavage site of *MrLAC5* using the 5′-RLM-RACE assay. It consistent with the cleavage site of other plants (Zhang et al., 2017; Pan et al., 2018), indicating that miR408 is functionally conserved in *M. rutheni*ca. As has been evidenced previously, Cu miRNAs are upregulated in the absence of Cu, which can mediate the post-transcriptional downregulation of transcripts encoding Cu proteins (Burkhead et al., 2009). Additionally, it has been shown that the Cu miRNAs tightly participate in regulating various processes of plant growth and development (Pilon, 2017). miR397 and miR857 have been reported to regulate lignin biosynthesis in *Arabidopsis* via targeting several laccase genes (Wang et al., 2014; Zhao et al., 2015). miR397-*OsLAC* can influence the plant architecture and yield of rice (Zhang et al., 2013). We have also previously reported that the miR397-*MrLAC17* module affects lignin synthesis in *M. ruthenica* (Zhang et al., 2022b). In the current work, we proposed that modulating miR408-*MrLAC5* expression pattern may enhance the photosynthetic efficiency and thus increase the yield in *M. ruthenica*.

## 4 Materials and methods

### 4.1 Plant materials preparation

*M. ruthenica* ‘Zhilixing’ (hereinafter referred to as ZLX), *M. ruthenica* ‘Mengnong No.1’ (MN No.1) and *M. ruthenica* ‘Mengnong No.2’ (MN No.2) were used in this study. ZLX was registered as a new variety by China Forage Variety Registration Committee in 1993 (Registration No.: 130). MN 1 and MN 2 were approved by the Forage Variety Resources Registration Committee of Inner Mongolia Autonomous Region in 2019 (Registration No.: 003–2019) and 2020 (Registration No.: 020–2020), respectively. Before the experiment, all the plants were cultivated (111°23‘46“E, 40°31′17″N) for more than 10 years, showing stable phenotypic characteristics. The seeds were sown at an base (111°22′30″E, 40°41′30″N) at August end, 2018 (Zhang et al., 2022b). Leaves at the early flowering stage were sampled in 2020. The collected samples were then immediately frozen with liquid nitrogen, followed by storage (−80°C) for the next-step experiment.

### 4.2 Leaf phenotype and leaf anatomy

The 3-month-old plants were measured for leaf morphology. A total of 20 leaves were selected (10 cm below the top of each plant). Leaf length and width (the widest position) of the middle leaflet of a ternately compound leaf were determined using a vernier caliper, followed by measuring leaf area with mesh paper with an accuracy of 1 mm (1 mm^2^ per grid).

The anatomical structure of leaves at the early-flowering stage was observed. Specifically, following procedures published previously (Zhang et al., 2022b), leaves at the top of each plant (sampled in four directions: east, west, north, and south) were fixed in formalin-glacial acetic acid −70% ethyl alcohol (FAA, 1:1:18, V/V/V) solutionand dehydrated with xylene and gradient (100%-100%–75%) ethanol. Each step lasted 3–5 min. The samples were then dehydrated with a xylene-chloroform mixture (9:1, V/V). Each step lasted 20–30 min. Next, the transparent material was soaked in the mixture of V-paraffin: V-xylene = 1:1 for 6–8 h, and then transferred to 100% paraffin at 65°C for 8–15 h, and 100% paraffin was replaced every 2–3 h. The paraffin sections were dewaxed successively with 100% xylene (2 min, twice) and V anhydrous ethanol ∶V xylene = 1∶1(2 min), then with graded ethanol (anhydrous ethanol, 90% ethanol, 80% ethanol, 70% ethanol, 2 min each). After that, the sections underwent safranin O (at least 6 h)/Fast green (less than 30 s) staining, and dehydration was carried out in combination with 95% and anhydrous ethanol washing tablets (3–4 times). Finally, the sealed samples were observed under an optical microscope.

### 4.3 Chlorophyll concentration

Leaf samples were collected from 10 plants for each cultivar in four directions (east, west, south, and north). Leaves (0.2 g fresh weight, FW) were prepared. Chl was extracted utilizing 10 mL (V) of 80% acetone away from light until the leaf become white. The optical density (OD) value at 663 nm (*OD*_*663*_), 645 nm (*OD*_*645*_), and 470 nm (*OD*_*470*_) was measured using Alan’s method for detecting Chl a and Chl b (Alan, 1994). Chl t and Car were calculated as follows.

Chlorophyll concentration (mg^**.**^L^-1^): (1)–(4).

$$Chl\;a=12.2\times {OD}_{663}-2.81\times {OD}_{645}$$ (1)

$$Chl\;b=20.13\times {OD}_{645}-5.03\times {\text{OD}}_{663}$$ (2)

Chl t=Chl a+Chl b (3)

$$Car=\left(1000\times {OD}_{470}-3.27\times Chl\;a-104\times Chl\;b\right)/229$$ (4)

$$\text{Chlorophyll content}\left(\text{mg}.g^{-1}\right)=\left(\text{Chlorophyll concentration}\times \text{Extraction volume}\times D\text{ilution ratio}\right)/\text{FW}$$ (5)

### 4.4 Photosynthetic characteristics

According to the slightly modified method of Li et al. (Li et al., 2014), the photosynthetic light response curves of the expanded leaves in 3 *M. ruthenica* cultivars were measured. The photosynthetic active radiation (PAR) in leaf chamber of Li-6400 photosynthesis system was adjusted by applying a red and blue light source; the light intensity was set as 0, 50, 100, 150, 200, 400, 600, 800, 1000, 1200, 1500, and 2000 μmol m^-2^.s^-1^. The Pn, stomatal conductance (Gs), intercellular CO_2_ concentration (Ci), and transpiration rate (Tr) of three cultivars under different light intensities were measured from 9:00 to 11:00 a.m. Test material selection and repeated setting in light response curve determination were similar to that in Chl concentration determination. The optical response curve was fitted by the Farquhar model (Farquhar et al., 2004), and the calculation formula was as follows:

$$P_{n}=\frac{light\times AQE+P_{\max}-\sqrt{{\left(light\times AQE+P_{\max}\right)}^{2}-4light\times AQE{\times P}_{\max}}}{2K}-R_{day}$$ (6)

In the above formula, AQE is the apparent quantum efficiency, R_day_ is the dark respiration rate, K is the bending angle of the light response curve, and P_max_ is the maximum Pn. The LSP and LCP parameters were calculated using the Richardson method (Richardson and Berlyn, 2002).

### 4.5 Transcriptome sequencing and data analysis

RNA-seq analysis was performed on Illumina Novaseq™ 6000 (Lianchuan Biotechnology Co., Ltd., Hangzhou, China) using high-quality RNA from three biological samples of each cultivar, following a set protocol. For obtaining the information on reads, Hisat2 (Kim et al., 2019) was used to perform reference genome alignment on the valid data after preprocessing. Genome information of *M. ruthenica* (accession WNNG0000000) was obtained by Wang (Wang et al., 2021).

Fragments per kilobase million (FPKM) values served for gene expression normalization. DE-Seq software was employed for identifying differentially expressed genes (DEGs) among different groups (Kvam et al., 2012). Genes with a corrected *p*-value of <0.05 and |log2 FC| of >1 were considered as DEGs. Afterwards, Gene Ontology (GO) (http://www.geneontology.org) and Kyoto Encyclopedia of Genes and Genomes (KEGG) (https://www.genome.jp/kegg/) enrichment analyses were carried out on all DEGs; significant enrichment was identified with *p* ≤ 0.05 as a threshold in hypergeometric tests. Visualization of the enrichment terms was processed using REVIGO software (Supek et al., 2011).

### 4.6 Small RNAs sequencing and miRNA basic analysis

Small RNA libraries of 9 *M. ruthenica* samples (three cultivars, each with three biological replicates) were constructed using TruSeq Small RNA Sample Prep Kits (Illumina, San Diego, United States), which were then sequenced by Illumina Hiseq 2500 (LC Sciences, United States) with single-end (SE50) sequencing (1 × 50 bp). Clean reads were obtained using an in-house tool designated ACGT101-miR (LC Sciences). Subsequently, unique sequences containing 18–25 bases were mapped to miRBase 22.0 by a BLAST search for miRNAs identification. miRNA family classification, miRNA frequency in other species, miRNA precursor number in different species and miRNA base preference were statistically analyzed. The specific calculation method was referred to the previous report (Zhang et al., 2022b).

### 4.7 Degradome libraries construction and analysis

The construction of degradome libraries was simplified and optimized based on previous report (Ma et al., 2010). A NanoDropND-1000 (NanoDrop, Wilmington, DE, United States) was employed for the quantification of total RNA and the purity. After that, Poly (A) RNA was purified from ∼20 μg RNA of three cultivar groups (three biological replicates per cultivar were mixed as one sample) using poly-T oligo-attached magnetic beads. The first strand cDNA was synthesized using a 3-adapter random primer. Next, PCR was used for amplifying degradome libraries; Illumina Hiseq2500 (LC Bio, China) was applied for sequencing (single-end sequencing read with 1 × 50 bp length).

The CleaveLand 4.0 program was used for degradome analysis (Addo-Quaye et al., 2008). We identified mRNAs that matched the degradome sequence using oligomap short reading frame calibrator. GSTAr.pl, Generic Small RNA Transcript Aligner was used for predicting the mRNA sequences of target genes paired with the miRNA sequences. Common mRNA (the target gene of miRNA) was identified after combining the predicted miRNA-mRNA pairs with mRNA in the generated degradome density file. The predicted results were shown in T-plots.

### 4.8 Real-time quantitative reverse transcription PCR (qRT-PCR) analysis

Total RNA extraction and cDNA synthesis from leaves of three *M. ruthenica* cultivars were performed using RNAprep Pure Plant Kit (DP441) and Hi-DNAsecure Plant Kit (DP350), respectively.

Specific steps were performed according to the manufacturer’s instructions. *EasyScript* ^®^ One-Step gDNA Removal and cDNA Synthesis SuperMix Kit (AE311, Beijing TransGen Biotech Co., LTD.) was used to perform reverse transcription. The oligo (dT)18 primer was used for target mRNAs, and specific stem-loop RT primers for miRNA. Following the previous method (Zhang et al., 2022b), TB Green^®^ Premix Ex Taq™ GC (Perfect Real Time) and a CFX Connect Real-Time PCR Detection System (Bio-RAD, United States) were used for qRT-PCR analysis. *MrACTIN* and U6 snRNA served as an internal reference for mRNA and miRNA expression analysis, resectively. All the primers were shown in Supplementary Table S10.

### 4.9 Validation of target cleavage site by 5′-RLM-RACE

miRNA target validation was performed using the RNA ligase-mediated of 5′ cDNA rapid amplification according to the method the manufacturer’s instructions for Invitrogen™ FirstChoice™ RLM-RACE Kit (AM1700). Total RNA with an adaptor was converted to cDNA and PCR reaction was performed using GeneRacer 5 primer and gene-specific reverse primer (Supplementary Table S10). The amplified products were followed by a new round of PCR reaction using GeneRacer 5-nested primer and nest-specific primer for the same gene (Supplementary Table S10). The final PCR target product was cloned using the Invitrogen™ Kit TOPO™ TA Cloning™. At least 10 clones of each PCR product were sequenced.

## 5 Conclusion

In conclusion, we identified key miRNAs and their target genes that might involve in high photosynthetic efficiency in *M. ruthenica* through small RNA, transcriptome, and degradome sequencing. However, the complicated miRNAs-mediated regulatory network remains to be further clarified. Currently, the key modules discerned in this inquiry not only furnish a benchmark for breeding programs aimed at enhancing photosynthetic efficiency in *M. ruthenica* but also offer novel insights into the complex mechanisms governing photosynthetic efficiency. Moreover, these findings contribute to the strategic bio-design and breeding of leguminous forage crops.

## Data Availability

The datasets presented in this study can be found in online repositories. The raw sequencing data can be found at https://www.ncbi.nlm.nih.gov/sra/PRJNA962259.
