# Supplementary figures and images for: Characterization of the microRNA408-LACCASE5 module as a regulatory axis for photosynthetic efficiency in Medicago ruthenica: implications for forage yield enhancement
Source: Front Genet. 2023 Nov 28;14:1295222. doi: 10.3389/fgene.2023.1295222 (PMC10713734; doi:10.3389/fgene.2023.1295222)

**Figure S2.** The expression level of *SCL6* in three *M. ruthenica* cultivars was detected

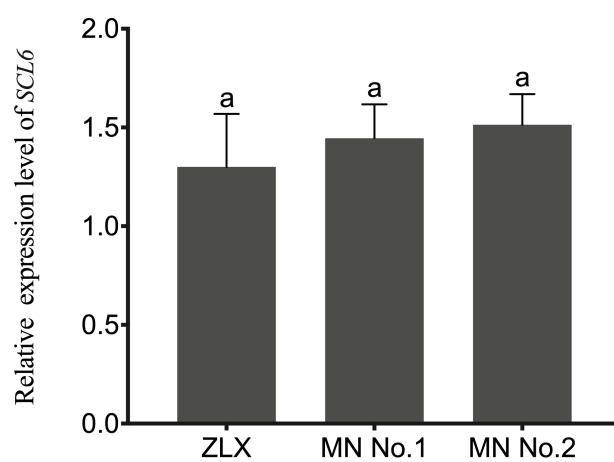

Supplement: Supplementary file 1 [file DataSheet2.PDF]

**Figure S1.** DEGs in different groups were identified

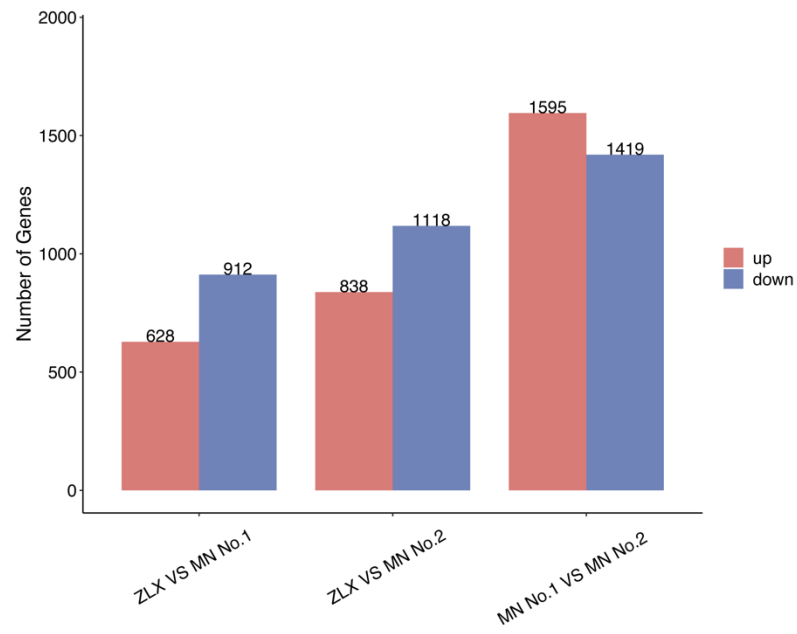

Supplement: Supplementary file 7 [file DataSheet1.PDF]
