# Supplementary material for: Characterization of the microRNA408-LACCASE5 module as a regulatory axis for photosynthetic efficiency in Medicago ruthenica: implications for forage yield enhancement
Source: Front Genet. 2023 Nov 28;14:1295222. doi: 10.3389/fgene.2023.1295222 (PMC10713734; doi:10.3389/fgene.2023.1295222)
Supplement: Supplementary file 3 [file Table1.DOCX]

**Table S1.** Comparison of yield and yield-related traits of different plant-type *M.ruthenica*

| **Year of growth** | **Cultivar** | **Natural plant height (cm)** | **Absolute plant height (cm)** | **Number of branches** | **Number of leaves** | **Hay yield (kg/hm^2^)** |
| --- | --- | --- | --- | --- | --- | --- |
| The second year | ZLX  MN No.1  MN No.2 | 65.25±5.67a  30.31±2.11b  34.50±1.86b | 74.55±0.77c  97.53±3.34a  89.67±2.65b | 29.05±0.33ab  26.84±4.54b  34.70±7.89a | 88.64±9.81b  94.75±15.32b  184.61±37.37a | 4691.42±133.55a  5517.81±482.18a  4798.67±292.74a |
| The third year | ZLX  MN No.1  MN No.2 | 80.33±3.32a  49.60±7.21b  43.00±0.98b | 87.73±2.32b  102.40±6.33a  92.90±4.62b | 45.00±3.91b  47.60±9.41b  55.50±1.68a | 129.75±14.50b  132.07±21.65b  230.17±33.37a | 4926.82±171.49c  6091.52±116.33a  5573.68±377.36b |
| The fourth year | ZLX  MN No.1  MN No.2 | 82.33±2.06a  58.20±1.67b  57.27±3.24b | 90.48±1.32c  124.50±5.36a  98.64±3.45b | 47.30±1.67b  58.00±4.65a  63.00±2.91a | 127.08±29.02b  131.13±21.92b  264.00±36.68a | 5515.47±462.77b  7121.01±627.85a  6005.72±381.59b |
| Three-year average | ZLX  MN No.1  MN No.2 | 75.97±9.33a  46.03±10.98b  44.92±11.51b | 84.25±8.51c  108.14±14.37a  93.74±4.54b | 40.45±9.94b  44.15±15.86b  51.07±14.66a | 115.16±23.00b  119.32±21.81b  226.26±39.84a | 5044.57±424.46b  6243.45±812.32a  5459.36±611.59b |

Note: In the same year, the same column with completely different lowercase letters indicates significant differences (*P*<0.05).
