# Supplementary material for: Characterization of the microRNA408-LACCASE5 module as a regulatory axis for photosynthetic efficiency in Medicago ruthenica: implications for forage yield enhancement
Source: Front Genet. 2023 Nov 28;14:1295222. doi: 10.3389/fgene.2023.1295222 (PMC10713734; doi:10.3389/fgene.2023.1295222)
Supplement: Supplementary file 6 [file Table2.DOCX]

**Table S2.** Correlation between plant type characters and yield and its related traits in *M.ruthenica*

| **Index** | **Natural plant height (cm)** | **Absolute plant height (cm)** | **Number of branches** | **Number of leaves** | **Hay yield (kg/hm^2^)** | |
| --- | --- | --- | --- | --- | --- | --- |
| Leaf length  Leaf width  Ratio of leaf length to leaf width  Leaf area | 0.42585  0.85974  -0.72010  0.69613 | 0.64894  0.96915  -0.51213  0.98959* | 0.86245  0.11604  -0.88227  -0.64424 | 0.91167  0.45497  -0.99102  -0.89454 | | 0.97298  0.99935**  -0.66563  0.98914* |

Note：* indicates a significant correlation at the level of 0.05, and ** indicates a significant correlation at the level of 0.01.
