# Supplementary material for: Characterization of the microRNA408-LACCASE5 module as a regulatory axis for photosynthetic efficiency in Medicago ruthenica: implications for forage yield enhancement
Source: Front Genet. 2023 Nov 28;14:1295222. doi: 10.3389/fgene.2023.1295222 (PMC10713734; doi:10.3389/fgene.2023.1295222)
Supplement: Supplementary file 12 [file Table10.DOCX]

**Table S10.** Primers used in this study

| **Primer name** | **Sequence (5’ to 3’)** |
| --- | --- |
| *MrACTIN*-qRT-F | ATCCAGGCTGTCCTCTCCCT |
| *MrACTIN-qRT-R* | ACGAAGGATGGCATGTGGGA |
| U6 snRNA-F | ACAGAGAAGATTAGCATGGCCC |
| U6 snRNA-R | GACCATTTCTCGATTTGTGCG |
| Stem loop  miR408-RT | GTCGTATCCAGTGCAGGGTCCGAGGTATTCGCACTGGATACGACGCCAGG |
| mtr-miR408-3p-F | GGCATGCACTGCCTCTTC |
| mtr-miR408-3p-R | ATCCAGTGCAGGGTCCGAGG |
| Stem loop  miR171d-RT | GTCGTATCCAGTGCAGGGTCCGAGGTATTCGCACTGGATACGACTCTGAG |
| mtr-miR171d-F | GGCTATTGGCCTGGTTCA |
| mtr-miR171d-R | ATCCAGTGCAGGGTCCGAGG |
| Stem loop  miR398-RT | GTCGTATCCAGTGCAGGGTCCGAGGTATTCGCACTGGATACGACAGGGGT |
| mtr-mi398b-qRT-F | GGCTGTGTTCTCAGGTC |
| mtr-mi398b-qRT-R | ATCCAGTGCAGGGTCCGAGG |
| *HSR201*-qRT-F | GGCGTAAACTGGAACCATGC |
| *HSR201*-qRT-R | GTGCTCCTCGAGCCATTTCT |
| *PGR*-qRT-F | AAGGGTACAAATGGCGGTGT |
| *PGR*-qRT-R | ATCAGACCCGCACCTGATTG |
| *LAC5*-qRT-F | AAGGAACACGCTTCACTGCT |
| *LAC5*-qRT-R | CGAATTTCACGGGCGGTTTT |
| *SNUPN*-qRT-F | CTGGGATTACGCCGTTAGCA |
| *SNUPN*-qRT-R | GGATCGTCGCAGGTAGTCAG |
| *ACR8*-qRT-F | TCTTACCCAACCGTGAGTGC |
| *ACR8*-qRT-R | ACTGTAGCACCCTGATGCTTG |
| *At5g64700*-qRT-F | CACCGCAATGAGCATGGTTT |
| *At5g64700*-qRT-R | AGTTCCCAGGAGAATGCGAC |
| *MrLAC5-*5’*-*RACE*-*outer primer | GGTTGTGAATGGTTTAACATAGGAAGC |
| *MrLAC5-*5’*-*RACE*-*inner primer | CCTTGCTTTGGATGAATGATTAGAGCT |
